# Molecular Characterization of Colistin-Resistant Clinical *Acinetobacter baumannii* from Northern Greece: Phenotypic Colistin Susceptibility and *lpx/pmrCAB* Mutational Profiles

**DOI:** 10.3390/antibiotics15030318

**Published:** 2026-03-20

**Authors:** Dimitrios Karakalpakidis, Michaela-Eftychia Tsitlakidou, Michalis Paraskeva, Maria Nikoleta Mavidi, Maria Marinou, Kassandra Procter, Apostolos Beloukas, Christine Kottaridi

**Affiliations:** 1 Laboratory of General Microbiology, Department of Genetics, Development and Molecular Biology, School of Biology, Aristotle University of Thessaloniki, 54124 Thessaloniki, Greece; dimkarakal@gmail.com (D.K.); michaelatsitlakidou@gmail.com (M.-E.T.); michaelparaskv@gmail.com (M.P.); marianikoleta.mavidi@gmail.com (M.N.M.); 2 Laboratory of Molecular Microbiology and Immunology, Department of Biomedical Sciences, University of West Attica, 12243 Athens, Greece; dml24021@uniwa.gr (M.M.); kprokter@uniwa.gr (K.P.); abeloukas@uniwa.gr (A.B.)

**Keywords:** *Acinetobacter baumannii*, colistin resistance, Lipopolysaccharide (LPS) genes, *pmrCAB* operon, MLST, XDR/PDR isolates, sequence type (ST)

## Abstract

**Background:** *Acinetobacter baumannii* (*A. baumannii*) is a formidable nosocomial pathogen and is classified by the World Health Organization (WHO) as a critical-priority pathogen, owing to its rapid evolution into extensively drug-resistant (XDR) and pan-drug-resistant (PDR) strains. Colistin remains one of the last-resort therapeutic options, although resistance rates are increasing in endemic regions such as Greece. In this study, we investigated the molecular basis of colistin resistance and characterized the clonal backgrounds of clinical XDR/PDR *A. baumannii* isolates collected between January and June 2022 from two tertiary-care hospitals in Thessaloniki, Northern Greece. **Methods:** We analyzed forty non-duplicate XDR/PDR clinical isolates. Antimicrobial susceptibility was determined using the VITEK 2 system, broth microdilution, and gradient diffusion methods. The lipid A biosynthesis genes (*lpxA*, *lpxC*, *lpxD*) and the *pmrCAB* operon were amplified by PCR and sequenced for all isolates. A representative subset of strains (n = 10/40) underwent multilocus sequence typing (MLST) according to the Pasteur MLST scheme. **Results:** All isolates proved colistin-resistant (MIC ≥ 4 µg/mL), and 95% were classified as PDR. Sequence analysis revealed multiple nonsynonymous mutations in the *pmrCAB* operon, with the PmrB A226V substitution predominating and extensive amino-acid changes observed in PmrC. In contrast, *lpx* genes exhibited limited protein-level variation, limited to lineage-associated polymorphisms (LpxC N287D, LpxD E117K). A novel six-nucleotide insertion in *pmrB* was identified in one isolate. MLST demonstrated a predominance of ST2 (International Clone 2), with single representatives of ST115 (IC2) and ST1 (IC1). **Conclusions:** In this cohort from Northern Greece, chromosomal mutations in the *pmrCAB* operon, within a predominantly ST2/IC2 background, were strongly associated with colistin resistance. These findings underscore the urgent need for continued molecular surveillance and targeted infection-control measures to limit further spread of PDR *A. baumannii*.

## 1. Introduction

Antimicrobial resistance (AMR) is widely recognized as an escalating global health threat. In 2019, antimicrobial-resistant infections were estimated to be directly responsible for approximately 1.27 million deaths and associated with nearly 4.95 million deaths worldwide annually [1,2]. Among multidrug-resistant (MDR) Gram-negative bacteria, *Acinetobacter baumannii* has been classified by the WHO as a “critical-priority” pathogen, reflecting the rapid emergence of extensively drug-resistant (XDR) and pandrug-resistant (PDR) strains [3]. Colistin, used as a last-resort therapeutic option against severe infections, has shown declining effectiveness due to the rise in resistance [2]. Colistin resistance in *A. baumannii* is multifactorial and linked to diverse molecular mechanisms. Alterations in genes involved in lipopolysaccharide (LPS) biosynthesis (*lpxA*, *lpxC*, *lpxD*) can disrupt the integrity of the outer membrane, thereby reducing colistin binding capacity [4,5]. Similarly, changes in the PmrAB two-component regulatory system lead to phosphoethanolamine modifications to lipid A, decreasing susceptibility to the drug [4,5]. In addition, although rare, other mechanisms, such as plasmid-mediated *mcr* genes or regulatory pathway alterations, have been reported [6,7].

Multilocus sequence typing (MLST) is widely used for population-level characterization of *A. baumannii* and relies on sequencing internal fragments of seven housekeeping genes, followed by the assignment of isolates to sequence types (STs) based on their allelic profiles [8,9,10]. Two MLST schemes have been developed for *A. baumannii*, the Oxford and the Pasteur schemes, which use different sets of loci and therefore generate independent sequence type (ST) designations [9,10]. The Oxford scheme provides higher discriminatory power, but several of its loci are affected by homologous recombination and the presence of paralogous copies, which may complicate phylogenetic interpretation [11,12,13]. In contrast, the Pasteur scheme is based on more conserved loci and tends to define clonal groupings that better reflect monophyletic lineages [11,12,13,14]. For this reason, and due to its wide use in epidemiological surveillance studies, the Pasteur MLST scheme was selected for the analysis of the isolates included in this study.

According to the European Centre for Disease Prevention and Control (ECDC) data, Greece ranks among the European countries with the highest prevalence of MDR Gram-negative bacteria, with *A. baumannii* being a major cause of hospital-acquired infections [15]. The most recent reports show that resistance to carbapenems and other antimicrobials remains alarmingly high in Greek hospitals [4,15]. Based on 61 isolates reported to GLASS in 2023, colistin resistance reached 48%; however, this estimate is derived from a limited dataset [16,17,18]. Despite the severity of the situation, molecular data explaining colistin resistance in Greek *A. baumannii* isolates remain limited.

This study seeks to address this knowledge gap by molecular characterization of clinical XDR and PDR *A. baumannii* isolates recovered from two hospitals in Thessaloniki, northern Greece. The analysis focuses on mutations affecting the LPS biosynthesis genes *lpxA*, *lpxC*, and *lpxD*, as well as the *pmrCAB* operon involved in colistin resistance. In parallel, MLST is applied to define the clonal background of the isolates and to assess the distribution of STs among colistin resistance. This combined approach links the genetic basis of resistance with the local epidemiological framework that supports the persistence of high-risk *A. baumannii* lineages in the hospital settings.

## 2. Results

### 2.1. Isolate Collection

In total, 40 XDR and PDR *A. baumannii* isolates were analyzed, each representing a single hospitalized patient. Twenty-seven isolates originated from Hippokration General Hospital and thirteen from G. Papanikolaou General Hospital. Nearly half of the isolates were derived from ICU patients (n = 19), with additional cases originating from the Internal Medicine Unit (n = 7), the Respiratory Failure Unit (RFU, n = 3), and the Plastic Surgery Unit (PSU, n = 3). The remaining eight isolates were obtained from the High Dependency Unit (HDU, n = 2), the Surgical Ward (Surg, n = 2), the Cardiology Ward (Cardio, n = 1), the Obstetrics and Gynecology Unit (ObGyn, n = 1), the Neurology Ward (Neuro, n = 1), and the Nephrology Ward (Nephro, n = 1). The study cohort included 23 male and 17 female patients.

### 2.2. Colistin Resistance and Antimicrobial Susceptibility

Of the 40 *A. baumannii* isolates analyzed, 38 (95%) exhibited a pandrug-resistant (PDR) phenotype, while the remaining two were classified as extensively drug-resistant (XDR). Colistin susceptibility was evaluated using the reference broth microdilution (BMD) method as well as the gradient diffusion (E-test) method. Both methods consistently confirmed resistance in all isolates, with minimum inhibitory concentration (MIC) values ≥ 4 µg/mL, according to current clinical breakpoints. The results obtained were fully concordant between the two testing methods and were also in agreement with the resistance profiles previously reported by the BioMérieux VITEK 2 automated system in the participating hospitals (Table S1).

### 2.3. Multilocus Sequence Typing

MLST was performed on 10 of the 40 clinical isolates, selected to represent the previously identified PFGE clusters [4], which were defined based on ≥85% similarity of banding patterns using the Dice coefficient and unweighted pair group method with arithmetic mean (UPGMA). We acknowledge that selecting isolates based on these clusters may introduce some bias in clonal distribution; however, this approach was chosen to ensure representation of the major circulating clones in our collection. Among the analyzed isolates, eight were assigned to sequence type ST2, one to ST115, and one to ST1. According to the Pasteur MLST scheme, ST2 and ST115 belong to International Clone 2 (IC2), whereas ST1 is classified within International Clone 1 (IC1). Overall, 9 of the 10 typed isolates belonged to IC2, while only a single isolate was associated with IC1. The clonal predominance is inferred from a subset and cannot represent the entire collection.

### 2.4. Mutations in the lpx and pmr Genes

In the *lpxA* gene, several nucleotide polymorphisms were identified; none resulted in non-synonymous substitutions in the examined sequences. In the *lpxC* gene, the nucleotide substitution A859G, corresponding to an N287D amino acid change, was identified in 36/40 (90%) of the sequenced isolates. Similarly, in the *lpxD* gene, the G349A mutation, resulting in an E117K substitution, was detected in 30/40 (75%) of the analyzed sequences. No amino acid-altering substitutions were identified in *pmrA* upon analysis of the aligned and translated sequences. In *pmrB*, analysis of the sequenced region identified five non-synonymous mutations. The most prevalent was the A226V substitution, detected in the majority of isolates. Additional substitutions, including K179M, E210D, and A275E, were identified in a small number of isolates. A six-nucleotide insertion (821_822insACGATT) was observed in a single isolate, resulting in the insertion of two amino acids (Leu–Ala) in the PmrB protein.

**Figure 1:**
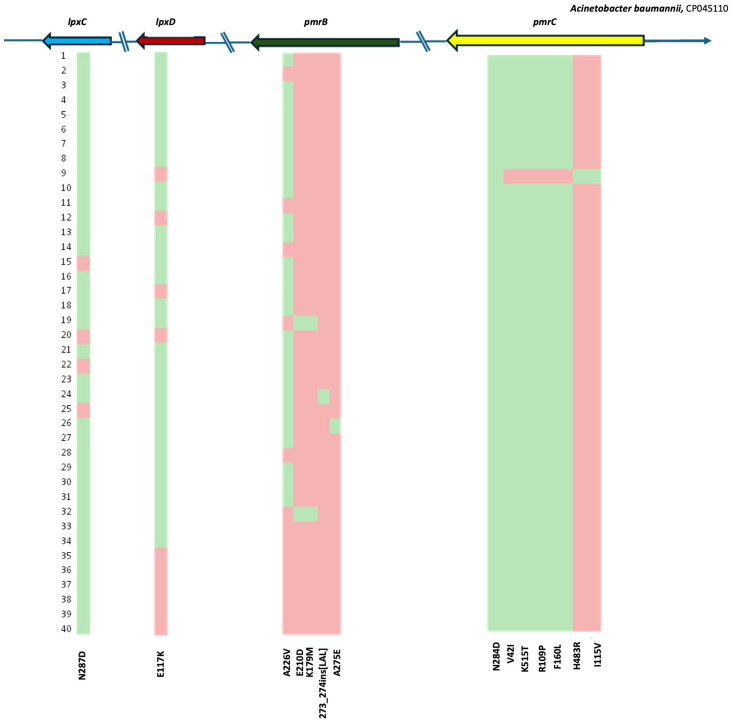
Heat map showing the distribution of mutations in the *lpxC*, *lpxD*, *pmrB*, and *pmrC* genes of colistin-resistant *A. baumannii* clinical isolates, based on the reference genome CP045110. Arrows indicate gene orientation. Green denotes mutated regions, while red indicates wild-type sequence. Numbers on the left represent the isolates’ numbering.

**Figure 2:**
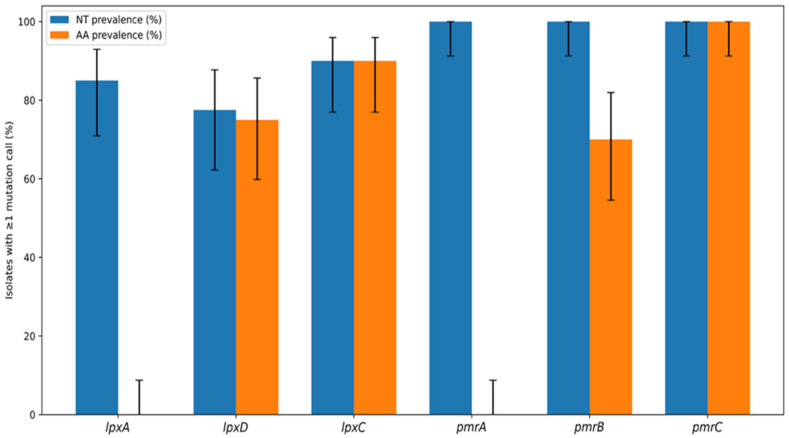
Gene-level mutation prevalence with 95% Wilson confidence intervals. Bar plots show the percentage of isolates with ≥1 mutation call (the presence of at least one mutation) at the nucleotide (NT) level and the corresponding amino-acid (AA) level for *lpxA*, *lpxD*, *lpxC*, *pmrA*, *pmrB*, and *pmrC*. Error bars indicate 95% Wilson confidence intervals for each proportion.

In *pmrC*, multiple amino acid substitutions were detected. All isolates carried the N284D substitution. All isolates carried the N284D substitution in *PmrC*. Given its universal presence across the collection, this substitution may reflect lineage-associated polymorphism linked to the predominant clonal background. Five substitutions (V42I, R109P, F150L, A254S, and K515T) were present in 38 of the 40 isolates. However, several substitutions were isolate-specific: I115V was detected only in two isolates, I326T in one isolate and H483R in another one isolate (Figure 1). The prevalence of amino acid substitutions across the analyzed genes (*lpxA*, *lpxD*, *lpxC*, *pmrA*, *pmrB*, and *pmrC*) is presented in Figure 2, while the mutation frequency per sequence type is presented at Table S2.

**Figure 3:**
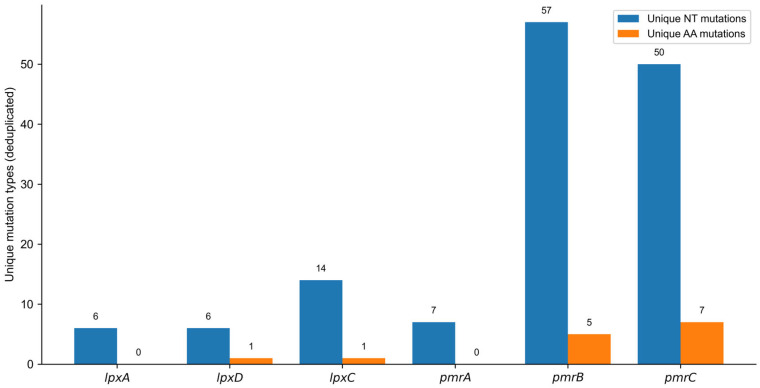
Unique mutation burden at the nucleotide and amino acid levels across resistance-related loci. Bars display the number of deduplicated (unique) nucleotide (NT) mutations and unique amino acid (AA) substitutions identified per gene (*lpxA*, *lpxD*, *lpxC*, *pmrA*, *pmrB*, and *pmrC*). Values above the bars represent the counts.

Quantitative analysis revealed a striking concentration of mutational burden within the *pmrCAB* operon. *pmrC* exhibited the highest overall mutation load, followed by *pmrB*, whereas *pmrA* showed nucleotide variation without corresponding amino acid substitutions. In contrast, the *lpx* genes displayed moderate nucleotide variability with limited protein-level impact. Diversity analysis confirmed *pmrB* as the most heterogeneous locus at the nucleotide level, while *pmrC* showed the greatest amino acid diversity (Figure 3).

**Figure 4:**
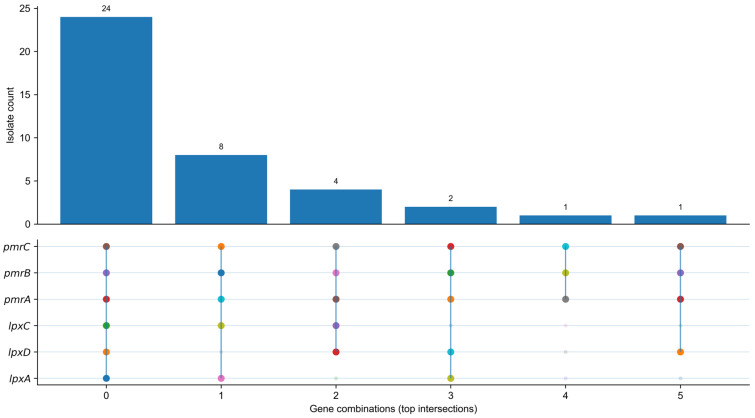
UpSet-style summary of gene-level co-occurrence of nucleotide (NT) mutations, where each isolate is scored “mutated” for a gene if ≥1 NT mutation entry is recorded. Bars indicate the number of isolates for each gene-combination (top intersections), and the dot matrix indicates which genes are included in each intersection. Colored dots indicate the presence of mutations in the corresponding genes; colors are used for visual distinction of mutation patterns and do not represent quantitative differences.

Analysis of gene-level mutations revealed that amino acid substitutions in the *pmrC* gene were detected in all samples. This finding is corroborated by the gene co-occurrence plot, in which *pmrC* appears consistently among the top-ranked combinations. Other genes, including *pmrB*, *pmrA*, *lpxC*, *lpxD*, and *lpxA*, exhibited mutations in a smaller subset of samples, with the most frequent combinatorial mutations involving *pmrC* and *pmrB* (n = 8), *pmrC* and *pmrA* (n = 4), and smaller combinations including the *lpx* genes (Figure 4, Table S3).

**Figure 5:**
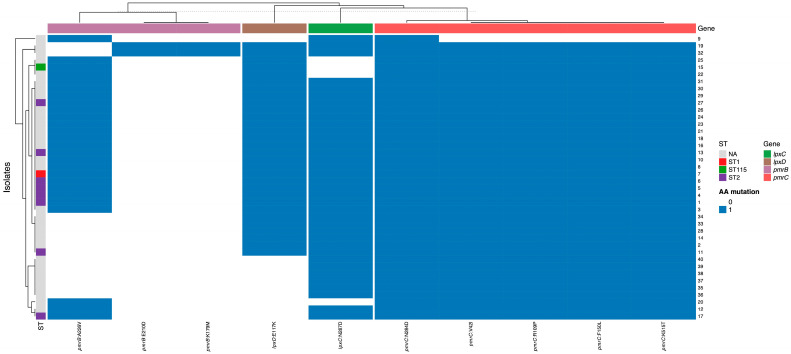
Mutation-profile clustering of amino-acid substitutions across isolates with ST overlay. Binary heatmap showing presence (blue) or absence (white) of amino-acid substitutions detected in the *lpx* and *pmr* loci. Columns represent AA substitutions (gene:mutation) filtered to those present in ≥2 isolates; rows represent isolates. Unsupervised hierarchical clustering was performed using Jaccard distance with complete linkage (dendrograms shown). Columns are grouped by gene (top color bar; *lpxC*, *lpxD*, *pmrB*, *pmrC*). The left annotation indicates MLST sequence type (ST) for typed isolates; isolates without MLST assignment are labeled NA.

To further visualize the distribution of amino-acid substitutions in relation to the clonal background of the isolates, a heatmap was constructed incorporating the detected mutations and the corresponding sequence types (STs) (Figure 5). The figure illustrates the presence of non-synonymous mutations across the analyzed *pmr* and *lpx* genes together with the ST classification of each isolate. This representation allows comparison of mutation patterns across isolates and provides a framework for examining their relationship with the predominant sequence types identified in the collection.

### 2.5. Comparison with Susceptible ST2 Genomes

Comparative analysis with publicly available colistin-susceptible ST2 genomes (BioProject PRJNA417158) was performed to differentiate lineage-associated polymorphisms from candidate resistance-associated mutations. The substitution LpxC N287D was also detected in the susceptible ST2 comparator genomes, suggesting that it likely represents lineage-associated variation rather than a resistance-specific change. In contrast, the substitutions identified in our isolate LpxD E117K, PmrB K179M, E210D, A226V, A275E, and PmrC V42I, R109P, I115V, F150L, N284D, H483R, K515T—were absent from the susceptible ST2 controls analyzed. Screening of 2386 publicly available *A. baumannii* genomes revealed no insertion events at *pmrB* codons 273–274. Notably, the ACGATT insertion corresponding to *pmrB* 273_274ins[LA] was not detected in any genome examined, suggesting that this mutation represents a previously unreported *pmrB* insertion.

## 3. Discussion

The emergence of colistin-resistant *A. baumannii* in Greek hospitals is concerning, as colistin remains one of the last therapeutic options for carbapenem-resistant infections [19,20]. All the isolates of the present study were colistin-resistant (MIC ≥ 4 µg/mL), and most exhibited a PDR phenotype. No plasmid-mediated *mcr* genes were detected, consistent with previous Greek studies showing that colistin resistance is primarily chromosomal, as *mcr-1* to *mcr-10* have not been identified in Greek isolates to date [4,18]. These findings indicate that resistance arises from intrinsic genomic mutations, as no plasmid-mediated *mcr* genes were detected in the present cohort [21,22,23]. National and regional surveillance data report increasing colistin resistance rates among Greek carbapenem-resistant *A. baumannii* (CRAB), reaching 27% in 2015 and ~33% in 2017 [18], while nearly half of isolates in multicenter Mediterranean ventilator-associated pneumonia (VAP) cohorts were PDR, including colistin resistance [19]. Together, these data highlight the sustained burden of colistin resistance in Greece and the need for continued surveillance as colistin use persists in ICU settings [20,24,25,26].

Our molecular findings align with previous Greek and international data. Multiple mutations were identified in the *pmrAB* two-component system and the lipid A biosynthesis pathway (*lpxACD*), both established mediators of colistin resistance. The PmrB A226V substitution predominated and has been consistently reported in Greek colistin-resistant isolates, where it is associated with activation of the *pmrAB* system and reduced colistin susceptibility [18]. Mutations in *pmrA* and *pmrB* upregulate *pmrC* expression, encoding a phosphoethanolamine transferase that modifies lipid A [27]. Lipid A modification by phosphoethanolamine (pEtN) has been documented in Greek resistant strains [18] and likely represents the dominant mechanism in our collection. Additional PmrB substitutions (K179M, E210D, and A275E) and synonymous *pmrA* polymorphisms were detected in selected isolates, consistent with reports linking multiple *pmrAB* mutations to elevated MICs (up to 64 µg/mL) [18]. A novel 6-nt insertion in *pmrB* resulted in a two–amino acid addition (Leu–Ala). Although rare, insertion events affecting *pmrB* have been described in colistin-resistant *A. baumannii*, including repeat insertions within PmrB and insertion sequence-mediated adaptive mutations emerging during colistin [28,29,30]. While the functional impact of this insertion remains uncertain, it should be considered a hypothetical mechanism. Structural alterations in the PmrB sensor domain could potentially influence activation of the *pmrCAB* operon and promote constitutive *pmrC* expression; however, this hypothesis would require confirmation through structural modeling approaches or complementation assays in future studies. The diversity of *pmrB* mutations in our isolates, including substitutions and an insertion, highlights the adaptive plasticity of *A. baumannii*, with distinct genetic routes converging on pEtN-mediated lipid A modification. Similar heterogeneity has been documented in Southeast Asia, where multiple PmrB and PmrC variants were identified across different clones [27], indicating convergent evolution toward disruption of the *pmrCAB* regulatory circuit. Overall, our data reinforce that PmrCAB-driven lipid A modification is the principal mechanism of colistin resistance in Greek *A. baumannii* [17,18]. In addition to regulatory alterations in *pmrA* and *pmrB*, structural variation in *pmrC* was observed in the present study. The N284D substitution was universal, while V42I, F150L, and K515T were detected in the vast majority of isolates. These variants have previously been reported in Greek and international collections and are considered lineage-associated polymorphisms rather than independent resistance determinants [17,18,27]. In contrast, R109P, H483R, and I115V have not been described in previous Greek studies nor documented in available international sequence databases. Interestingly, although alterations at residue 109 have been documented, published data primarily describe an R109H substitution, whereas the R109P variant observed in our collection has not been previously reported [31]. Moreover, substitutions such as N284D and I115V have been identified in colistin-susceptible isolates without a clear association with elevated MIC values [5,32]. Although *pmrC* exhibited the highest amino acid diversity in the present study, current evidence does not support an independent causal role of these variants in colistin resistance. Rather, they likely act in concert with *pmrB* mutations, reinforcing regulatory activation of the *pmrCAB* operon and phosphoethanolamine-mediated lipid A modification, as outlined in mechanistic reviews [5].

In contrast, no disruptive mutations were detected in *lpxA*, *lpxC*, or *lpxD*. Although polymorphisms were identified, most notably LpxC N287D and LpxD E117K, these variants have been described in both susceptible and resistant isolates [5,17] and are considered lineage-associated rather than causative unless combined with additional alterations [5,17,33]. While experimental knockouts of *lpxA*, *lpxC*, and *lpxD* confer high-level resistance through LPS loss [5,34,35] such mutants are rarely observed clinically [5,36,37,38], likely due to substantial fitness and virulence costs [5,39,40]. Consistently, our isolates retained modified LPS rather than exhibiting LPS deficiency, in agreement with sequencing and lipid A mass spectrometry studies from other regions [27,41,42]. These findings support that regulatory lipid A modification via PmrC predominates in clinical resistance, whereas complete LPS loss is uncommon in hospital settings [27,38].

MLST, performed on a PFGE-selected subset of isolates [4], revealed predominance of ST2 and limited clonal diversity. The isolates selected for MLST were representative of the major PFGE clusters identified in the collection, allowing estimation of the predominant clonal background. Nevertheless, because only 10 of the 40 isolates were analyzed, this approach may not fully capture the genetic diversity of the entire collection, and a degree of selection bias cannot be excluded. Despite this limitation, the high proportion of IC2 among the tested isolates suggests lineage predominance, a distribution consistent with national data. In Greece, 80.9% of carbapenem-resistant *A. baumannii* belonged to IC2 (all ST2) in 2015 [43] and 92.5% of colistin-resistant isolates collected during 2015–2017 were IC2, all carrying PmrB A226V [18]. ECDC surveillance likewise documented increasing colistin resistance in Greece from 1% (2012) to 27.3% (2015), with high rates among VAP isolates [18]. Similar IC2 predominance has been reported in Sicily (ST2/IC2 outbreak) [44], Serbia (76.7% ST2) [45], and Cairo (mainly ST2 within CC2) [46] supporting the association between IC2 and the dissemination of XDR/PDR strains. All ST2 isolates in our study carried PmrB A226V [5], along with LpxC N287D and LpxD E117K, consistent with lineage-associated polymorphisms described in Greek IC2 strains [17]. ST115 (CC2) was detected once and is closely related to ST2 [47], while ST1 appeared once, reflecting the lower prevalence of IC1 in Greece [43].

In summary, mutations detected in the *pmrCAB* and *lpx* loci in our isolates involve genes previously implicated in chromosomal mechanisms of colistin resistance in *A. baumannii*, particularly those affecting lipid A modification pathways [5,23,42]. However, because whole-genome sequencing was not performed, additional resistance determinants—such as mutations in other regulatory loci or the involvement of efflux systems—cannot be excluded [47]. The predominance of IC2/ST2 among the analyzed isolates is consistent with international reports highlighting the role of this lineage in the dissemination of multidrug- and colistin-resistant *A. baumannii* in hospital environments [18,43,44]. The coexistence of colistin resistance with XDR/PDR phenotypes further underscores the clinical relevance of this clone. Continued molecular surveillance, infection control measures, and antimicrobial stewardship are therefore essential to limit the spread of these high-risk lineages [2,15]. Future studies incorporating whole-genome sequencing will be important to more comprehensively characterize the genetic determinants underlying colistin resistance in these strains.

## 4. Materials and Methods

### 4.1. Sampling and Selection Process

This study utilized clinical *A. baumannii* isolates previously collected during a prospective study in two tertiary-care hospitals in Thessaloniki, Greece. Relevant microbiological data were obtained from laboratory databases to ensure completeness and accuracy. The isolates originated from Hippokration General Hospital (approximately 900 beds) and G. Papanikolaou General Hospital (approximately 750 beds). Ethical approval was obtained from the Scientific Councils of both hospitals (Hippokration: 9336/24-2-2022, G. Papanikolaou: 557/14-4-2022). Regarding the type of clinical specimens, the primary source of isolation was aerobic blood cultures (40.0%), followed by respiratory samples (32.5%), including bronchial secretions and one bronchoalveolar lavage specimen. Isolates were also recovered from urine cultures (12.5%), while a smaller proportion originated from skin lesion samples (5.0%) and central venous catheter tip cultures (5.0%). Single isolates were obtained from a burn wound and a stool sample (2.5% each). The inclusion criteria were: (i) isolates recovered from hospitalized patients between 1 January and 30 June 2022; (ii) isolates obtained from all hospital wards; (iii) inclusion of only the first MDR *A. baumannii* isolate per patient; and (iv) isolates exhibiting both MDR and colistin resistance [4]. Subsequent antimicrobial susceptibility testing revealed that a large proportion of these isolates were classified as PDR. The exclusion criteria were: (i) duplicate isolates obtained from the same patient; (ii) isolates not meeting the MDR and colistin-resistance criteria; and (iii) isolates collected outside the defined study period. The isolates were analyzed to investigate the molecular mechanisms underlying colistin resistance.

### 4.2. Antimicrobial Susceptibility Testing

Antimicrobial susceptibility profiles were evaluated in duplicate using the VITEK 2 automated platform (bioMérieux, Marcy l’Étoile, France). The activity of colistin was further evaluated using the reference broth microdilution method recommended by EUCAST. Minimum inhibitory concentration (MIC) values were determined by visual inspection following incubation. Colistin MICs ranged from 4 to 32 mg/L (MIC50, 8 mg/L; MIC90, 16 mg/L), consistent with the resistance profiles previously reported by the BioMérieux VITEK 2 automated system in the study’s hospitals. Growth and sterility controls were included in each assay to ensure the reliability of the results. The findings were additionally verified using gradient diffusion MIC strips (Liofilchem, Roseto degli Abruzzi, Italy) [48,49,50].

All results were interpreted according to the clinical breakpoints defined by the European Committee on Antimicrobial Susceptibility Testing (EUCAST). Based on these criteria, isolates were classified as MDR, XDR, and PDR, following established international definitions [51].

### 4.3. Multilocus Sequence Typing (MLST)—Pasteur Scheme

Genomic DNA was extracted using the PureLink™ Genomic DNA Mini Kit (Thermo Fisher Scientific, Waltham, MA, USA), according to the manufacturer’s instructions. MLST was performed according to the Pasteur scheme for *A. baumannii*. Ten of the 40 clinical isolates were selected for MLST analysis based on previously reported PFGE clusters [4], representing the major clonal groups in the collection. Internal fragments of the seven housekeeping genes *cpn60*, *fusA*, *gltA*, *pyrG*, *recA*, *rpoB*, and *rplB* were amplified by conventional PCR using primers obtained from the PubMLST database, following previously described reaction conditions (Table S4) [52]. Allele numbers and sequence types were assigned using the PubMLST online database according to the Pasteur MLST scheme [53].

### 4.4. Analysis of lpx Genes and the pmrCAB Operon

The *lpxA*, *lpxC*, and *lpxD* genes, as well as *pmrA*, *pmrB*, and *pmrC*, were amplified by conventional PCR using specific primers as previously described by Moffatt et al. and Beceiro et al. [38,42].

PCR products were subjected to bidirectional Sanger sequencing. The resulting sequences were compared with the reference genome of *A. baumannii* ATCC 19606 and analyzed using 4Peaks v1.8, Jalview v2.11.5.1, and MEGA X v10 software. The analysis included trimming, multiple sequence alignment using ClustalW v1.8 and MAFFT v7.490, and translation to the protein level. The sequences were examined for non-synonymous mutations and polymorphisms.

To distinguish lineage-associated polymorphisms from potential resistance-associated mutations, publicly available colistin-susceptible ST2 genomes were retrieved from BioProject PRJNA417158. Five susceptible isolates (GCF_002948475.1, GCF_002951015.1, GCF_002950975.1, GCF_002951435.1, and GCF_002951375.1), with their sequence type independently confirmed by in silico MLST using the Pasteur scheme, were used as susceptible ST2 comparators. The nucleotide sequences of the *lpxA*, *lpxC*, *lpxD*, *pmrA*, *pmrB*, and *pmrC* loci were extracted from these genomes and analyzed within the same reference framework used for the clinical isolates.

To assess whether the identified insertion corresponding to *pmrB* 273_274ins[LA] has been previously reported, publicly available *A. baumannii* assemblies were retrieved from the NCBI Assembly database using the NCBI Datasets CLI and screened locally. To ensure strict positional consistency, the screen was anchored to a full-length *pmrB* coding sequence extracted from the CP045110 reference framework. For each genome, the *pmrB* locus was recovered using a BLAST-based workflow (v2.16.0), aligned with MAFFT, and the region corresponding to codons 273–274 was specifically inspected for insertion events. Using this approach, we screened 2386 publicly available genomes, and no insertion was detected at this site in any screened genome. In particular, the ACGATT insertion corresponding to *pmrB* 273_274ins[LA] was not observed.

## 5. Conclusions

In conclusion, phenotypic and molecular analysis of colistin-resistant *A. baumannii* isolates from Northern Greece indicates that resistance in this collection is primarily associated with chromosomal variation in the *pmrCAB* pathway linked to phosphoethanolamine-mediated lipid A modification. Plasmid-mediated *mcr* genes were not detected, supporting the predominance of chromosomal rather than horizontally acquired resistance mechanisms. These findings highlight the importance of continued molecular surveillance of colistin resistance among high-risk *A. baumannii* lineages circulating in hospital settings. Overall, our results highlight the importance of combining phenotypic and molecular surveillance and demonstrate the translational potential of sequence-informed strategies to directly influence infection-control policies, optimize antimicrobial therapy, and support rapid diagnostic development.

This study has limitations. At first, clinical data were not available. In addition, colistin heteroresistance was not specifically investigated, as the study focused on isolates that were already phenotypically resistant to colistin according to EUCAST breakpoints. MLST was performed on a PFGE-selected subset of isolates, which may not fully represent the clonal distribution of the entire collection. Functional assays were not conducted to directly confirm the impact of specific mutations, including the novel *pmrB* insertion, on colistin resistance. A major limitation of this study is the absence of whole-genome sequencing (WGS), which would provide a more comprehensive characterization of resistance determinants, genomic context, and clonal relationships among the isolates. Finally, the study reflects isolates from a defined clinical and geographic setting, limiting broader generalization. Future studies incorporating genomic and functional analyses across multicenter cohorts would provide a more comprehensive understanding of resistance evolution.

## Data Availability

Data is contained within the article.

## Supplementary Materials

The following supporting information can be downloaded at: https://www.mdpi.com/article/10.3390/antibiotics15030318/s1, Table S1: The table presents the isolate identification number, clinical specimen type, hospital unit from which the sample was obtained, patient sex, hospital of origin, and the minimum inhibitory concentration (MIC) of colistin expressed in µg/ML; Table S2: Frequency of variable amino-acid substitutions (present in some but not all typed isolates), stratified by MLST sequence type (ST); Table S3: Nucleotide and amino acid mutations detected in *lpxA*, *lpxC*, *lpxD*, *pmrA*, *pmrB*, and *pmrC* genes among the 40 clinical isolates; Table S4: Primers (5′→3′) and expected PCR product sizes (bp) used for the amplification of the *lpxA*, *lpxD*, *lpxC*, *pmrA*, *pmrB*, *pmrB2*, *pmrC*, and *pmrC2* genes, as well as the seven housekeeping genes *cpn60*, *fusA*, *gltA*, *pyrG*, *recA*, *rplB*, and *rpoB* included in the MLST scheme for *A. baumannii*.

## References

[1] Murray C.J., Ikuta K.S., Sharara F., Swetschinski L., Robles Aguilar G., Gray A., Han C., Bisignano C., Rao P., Wool E. (2022). Global Burden of Bacterial Antimicrobial Resistance in 2019: A Systematic Analysis. Lancet.

[2] World Health Organization (2024). Global Antimicrobial Resistance and Use Surveillance System (GLASS) Report 2024.

[3] Prioritization of Pathogens to Guide Discovery, Research and Development of New Antibiotics for Drug-Resistant Bacterial Infections, Including Tuberculosis. https://www.who.int/publications/i/item/WHO-EMP-IAU-2017.12?utm_source=chatgpt.com.

[4] Karakalpakidis D., Papadopoulos T., Paraskeva M., Tsitlakidou M.-E., Vagdatli E., Katsifa H., Beloukas A., Kotzamanidis C., Kottaridi C. (2025). When the Last Line Fails: Characterization of Colistin-Resistant *Acinetobacter baumannii* Reveals High Virulence and Limited Clonal Dissemination in Greek Hospitals. Pathogens.

[5] Novović K., Jovčić B. (2023). Colistin Resistance in *Acinetobacter baumannii*: Molecular Mechanisms and Epidemiology. Antibiotics.

[6] Hematian A., Karami-Zarandi M., Rahdar H.A., Heidari H., Kazemian H. (2025). Investigation of Colistin Heteroresistance in Multidrug-Resistant *Acinetobacter baumannii* Clinical Isolates. New Microbes New Infect..

[7] Poirel L., Jayol A., Nordmanna P. (2017). Polymyxins: Antibacterial Activity, Susceptibility Testing, and Resistance Mechanisms Encoded by Plasmids or Chromosomes. Clin. Microbiol. Rev..

[8] Anwer R. (2024). Molecular Epidemiology and Molecular Typing Methods of *Acinetobacter baumannii*: An Updated Review. Saudi Med. J..

[9] Bartual S.G., Seifert H., Hippler C., Luzon M.A.D., Wisplinghoff H., Rodríguez-Valera F. (2005). Development of a Multilocus Sequence Typing Scheme for Characterization of Clinical Isolates of *Acinetobacter baumannii*. J. Clin. Microbiol..

[10] Diancourt L., Passet V., Nemec A., Dijkshoorn L., Brisse S. (2010). The Population Structure of *Acinetobacter baumannii*: Expanding Multiresistant Clones from an Ancestral Susceptible Genetic Pool. PLoS ONE.

[11] Hua X., Zhang L., He J., Leptihn S., Yu Y. (2020). Population Biology and Epidemiological Studies of *Acinetobacter baumannii* in the Era of Whole Genome Sequencing: Is the Oxford Scheme Still Appropriate?. Front. Microbiol..

[12] Din N.S., Rani F.M., Alattraqchi A.G., Ismail S., Rahman N.I.A., Cleary D.W., Clarke S.C., Yeo C.C. (2025). Whole-Genome Sequencing of *Acinetobacter baumannii* Clinical Isolates from a Tertiary Hospital in Terengganu, Malaysia (2011–2020), Revealed the Predominance of the Global Clone 2 Lineage. Microb. Genom..

[13] Gaiarsa S., Batisti Biffignandi G., Esposito E.P., Castelli M., Jolley K.A., Brisse S., Sassera D., Zarrilli R. (2019). Comparative Analysis of the Two *Acinetobacter baumannii* Multilocus Sequence Typing (MLST) Schemes. Front. Microbiol..

[14] Global Epidemiology|*Acinetobacter baumannii*. https://acinetobacterbaumannii.no/overview/global-epidemiology/.

[15] European Centre for Disease Prevention (2023). Control Antimicrobial Resistance Surveillance in Europe 2023 Annual Report.

[16] ResistanceMap. https://resistancemap.onehealthtrust.org/.

[17] Oikonomou O., Sarrou S., Papagiannitsis C.C., Georgiadou S., Mantzarlis K., Zakynthinos E., Dalekos G.N., Petinaki E. (2015). Rapid Dissemination of Colistin and Carbapenem Resistant *Acinetobacter baumannii* in Central Greece: Mechanisms of Resistance, Molecular Identification and Epidemiological Data. BMC Infect. Dis..

[18] Palmieri M., D’Andrea M.M., Pelegrin A.C., Perrot N., Mirande C., Blanc B., Legakis N., Goossens H., Rossolini G.M., van Belkum A. (2020). Abundance of Colistin-Resistant, OXA-23- and ArmA-Producing *Acinetobacter baumannii* Belonging to International Clone 2 in Greece. Front. Microbiol..

[19] Mantzana P., Protonotariou E., Kassomenaki A., Meletis G., Tychala A., Keskilidou E., Arhonti M., Katsanou C., Daviti A., Vasilaki O. (2023). In Vitro Synergistic Activity of Antimicrobial Combinations against Carbapenem- and Colistin-Resistant *Acinetobacter baumannii* and *Klebsiella pneumoniae*. Antibiotics.

[20] El-Sayed Ahmed M.A.E.G., Zhong L.L., Shen C., Yang Y., Doi Y., Tian G.B. (2020). Colistin and Its Role in the Era of Antibiotic Resistance: An Extended Review (2000–2019). Emerg. Microbes Infect..

[21] Jia H., Tong Q., Wang L., Wu Y., Li X., Li S., Kong Y., Zhang Y., Furlan J.P.R., Khine N.O. (2025). Silent Circulation of Plasmid-Borne Tet(X6) and BlaOXA-58 Genes in a Community-Acquired *Acinetobacter baumannii* Strain. Drug Resist. Updates.

[22] Vijayakumar S., Swetha R.G., Bakthavatchalam Y.D., Vasudevan K., Abirami Shankar B., Kirubananthan A., Walia K., Ramaiah S., Biswas I., Veeraraghavan B. (2024). Genomic Investigation Unveils Colistin Resistance Mechanism in Carbapenem-Resistant *Acinetobacter baumannii* Clinical Isolates. Microbiol. Spectr..

[23] Islam M.M., Jung D.E., Shin W.S., Oh M.H. (2024). Colistin Resistance Mechanism and Management Strategies of Colistin-Resistant *Acinetobacter baumannii* Infections. Pathogens.

[24] Li J., Nation R.L., Kaye K.S. (2019). Polymyxin Antibiotics: From Laboratory Bench to Bedside.

[25] Karakonstantis S., Kritsotakis E.I., Gikas A. (2020). Pandrug-Resistant Gram-Negative Bacteria: A Systematic Review of Current Epidemiology, Prognosis and Treatment Options. J. Antimicrob. Chemother..

[26] Karakonstantis S., Saridakis I. (2020). Colistin Heteroresistance in *Acinetobacter* Spp.: Systematic Review and Meta-Analysis of the Prevalence and Discussion of the Mechanisms and Potential Therapeutic Implications. Int. J. Antimicrob. Agents.

[27] Srisakul S., Wannigama D.L., Higgins P.G., Hurst C., Abe S., Hongsing P., Saethang T., Luk-in S., Liao T., Kueakulpattana N. (2022). Overcoming Addition of Phosphoethanolamine to Lipid A Mediated Colistin Resistance in *Acinetobacter baumannii* Clinical Isolates with Colistin–Sulbactam Combination Therapy. Sci. Rep..

[28] Lucas D.D., Crane B., Wright A., Han M.L., Moffatt J., Bulach D., Gladman S.L., Powell D., Aranda J., Seemann T. (2018). Emergence of High-Level Colistin Resistance in an *Acinetobacter baumannii* Clinical Isolate Mediated by Inactivation of the Global Regulator H-NS. Antimicrob. Agents Chemother..

[29] Charretier Y., Diene S.M., Baud D., Chatellier S., Santiago-Allexant E., Van Belkum A., Guigon G., Schrenzel J. (2018). Colistin Heteroresistance and Involvement of the PmrAB Regulatory System in *Acinetobacter baumannii*. Antimicrob. Agents Chemother..

[30] Lunha K., Thet K.T., Ngudsuntia A., Charoensri N., Lulitanond A., Tavichakorntrakool R., Wonglakorn L., Faksri K., Chanawong A. (2020). PmrB Mutations Including a Novel 10-Amino Acid Repeat Sequence Insertion Associated with Low-Level Colistin Resistance in Carbapenem-Resistant *Acinetobacter baumannii*. Infect. Genet. Evol..

[31] Zafer M.M., Hussein A.F.A., Al-Agamy M.H., Radwan H.H., Hamed S.M. (2023). Retained Colistin Susceptibility in Clinical *Acinetobacter baumannii* Isolates with Multiple Mutations in PmrCAB and LpxACD Operons. Front. Cell. Infect. Microbiol..

[32] Kim S.-H., Yun S., Park W. (2022). Constitutive Phenotypic Modification of Lipid A in Clinical *Acinetobacter baumannii* Isolates. Microbiol. Spectr..

[33] Nhu N.T.K., Riordan D.W., Nhu T.D.H., Thanh D.P., Thwaites G., Lan N.P.H., Wren B.W., Baker S., Stabler R.A. (2016). The Induction and Identification of Novel Colistin Resistance Mutations in *Acinetobacter baumannii* and Their Implications. Sci. Rep..

[34] Jovcic B., Novovic K., Dekic S., Hrenovic J. (2021). Colistin Resistance in Environmental Isolates of *Acinetobacter baumannii*. Microb. Drug Resist..

[35] Nurtop E., Baylndlr Bilman F., Menekse S., Kurt Azap O., Gönen M., Ergonul O., Can F. (2019). Promoters of Colistin Resistance in *Acinetobacter baumannii* Infections. Microb. Drug Resist..

[36] Lee J.Y., Chung E.S., Ko K.S. (2017). Transition of Colistin Dependence into Colistin Resistance in *Acinetobacter baumannii*. Sci. Rep..

[37] Moffatt J.H., Harper M., Adler B., Nation R.L., Li J., Boyce J.D. (2011). Insertion Sequence ISAba11 Is Involved in Colistin Resistance and Loss of Lipopolysaccharide in *Acinetobacter baumannii*. Antimicrob. Agents Chemother..

[38] Moffatt J.H., Harper M., Harrison P., Hale J.D.F., Vinogradov E., Seemann T., Henry R., Crane B., St. Michael F., Cox A.D. (2010). Colistin Resistance in *Acinetobacter baumannii* Is Mediated by Complete Loss of Lipopolysaccharide Production. Antimicrob. Agents Chemother..

[39] Beceiro A., Moreno A., Fernández N., Vallejo J.A., Aranda J., Adler B., Harper M., Boyce J.D., Bou G. (2014). Biological Cost of Different Mechanisms of Colistin Resistance and Their Impact on Virulence in *Acinetobacter baumannii*. Antimicrob. Agents Chemother..

[40] Karakonstantis S. (2020). A Systematic Review of Implications, Mechanisms, and Stability of In Vivo Emergent Resistance to Colistin and Tigecycline in *Acinetobacter baumannii*. J. Chemother..

[41] Qureshi Z.A., Hittle L.E., O’Hara J.A., Rivera J.I., Syed A., Shields R.K., Pasculle A.W., Ernst R.K., Doi Y. (2015). Colistin-Resistant *Acinetobacter baumannii*: Beyond Carbapenem Resistance. Clin. Infect. Dis..

[42] Beceiro A., Llobet E., Aranda J., Bengoechea J.A., Doumith M., Hornsey M., Dhanji H., Chart H., Bou G., Livermore D.M. (2011). Phosphoethanolamine Modification of Lipid A in Colistin-Resistant Variants of *Acinetobacter baumannii* Mediated by the PmrAB Two-Component Regulatory System. Antimicrob. Agents Chemother..

[43] Pournaras S., Dafopoulou K., Del Franco M., Zarkotou O., Dimitroulia E., Protonotariou E., Poulou A., Zarrilli R., Tsakris A., Skoura L. (2017). Predominance of International Clone 2 OXA-23-Producing-*Acinetobacter baumannii* Clinical Isolates in Greece, 2015: Results of a Nationwide Study. Int. J. Antimicrob. Agents.

[44] Agodi A., Voulgari E., Barchitta M., Quattrocchi A., Bellocchi P., Poulou A., Santangelo C., Castiglione G., Giaquinta L., Romeo M.A. (2014). Spread of a Carbapenem- and Colistin-Resistant *Acinetobacter baumannii* ST2 Clonal Strain Causing Outbreaks in Two Sicilian Hospitals. J. Hosp. Infect..

[45] Kabic J., Novovic K., Kekic D., Trudic A., Opavski N., Dimkic I., Jovcic B., Gajic I. (2023). Comparative Genomics and Molecular Epidemiology of Colistin-Resistant *Acinetobacter baumannii*. Comput. Struct. Biotechnol. J..

[46] Fam N.S., Gamal D., Mohamed S.H., Wasfy R.M., Soliman M.S., El-Kholy A.A., Higgins P.G. (2020). Molecular Characterization of Carbapenem/Colistin-Resistant *Acinetobacter baumannii* Clinical Isolates from Egypt by Whole-Genome Sequencing. Infect. Drug Resist..

[47] Yoon E.J., Courvalin P., Grillot-Courvalin C. (2013). RND-Type Efflux Pumps in Multidrug-Resistant Clinical Isolates of *Acinetobacter baumannii*: Major Role for AdeABC Overexpression and Aders Mutations. Antimicrob. Agents Chemother..

[48] Sacco F., Visca P., Runci F., Antonelli G., Raponi G. (2021). Susceptibility Testing of Colistin for *Acinetobacter baumannii*: How Far Are We from the Truth?. Antibiotics.

[49] Ezadi F., Ardebili A., Mirnejad R. (2018). Antimicrobial Susceptibility Testing for Polymyxins: Challenges, Issues, and Recommendations. J. Clin. Microbiol..

[50] Toutain P.L., Bousquet-Mélou A., Damborg P., Ferran A.A., Mevius D., Pelligand L., Veldman K.T., Lees P. (2017). En Route towards European Clinical Breakpoints for Veterinary Antimicrobial Susceptibility Testing: A Position Paper Explaining the VetCAST Approach. Front. Microbiol..

[51] Magiorakos A.P., Srinivasan A., Carey R.B., Carmeli Y., Falagas M.E., Giske C.G., Harbarth S., Hindler J.F., Kahlmeter G., Olsson-Liljequist B. (2012). Multidrug-Resistant, Extensively Drug-Resistant and Pandrug-Resistant Bacteria: An International Expert Proposal for Interim Standard Definitions for Acquired Resistance. Clin. Microbiol. Infect..

[52] Primers Used for MLST of *Acinetobacter baumannii* Complex (Pasteur Scheme)|PubMLST. https://pubmlst.org/primers-used-mlst-acinetobacter-baumannii-complex-pasteur-scheme.

[53] Multi-Locus Sequence Typing|PubMLST. https://pubmlst.org/multilocus-sequence-typing.

