# Supplementary material for: Molecular Characterization of Colistin-Resistant Clinical Acinetobacter baumannii from Northern Greece: Phenotypic Colistin Susceptibility and lpx/pmrCAB Mutational Profiles
Source: Antibiotics (Basel). 2026 Mar 20;15(3):318. doi: 10.3390/antibiotics15030318 (PMC13024636; doi:10.3390/antibiotics15030318)
Supplement: Supplementary file 1 [file antibiotics-15-00318-s001.zip › antibiotics-4191881-supplementary.pdf]

Isolate ID	Clinical specimen	Hospital unit	Patient sex	Hospital of origin	Colistin MIC (μg/mL)
1	Blood	ICU	Male	Hippokration	8
2	Blood	ICU	Male	Hippokration	32
3	Blood	Respiratory Failure Unit	Male	G. Papanikolaou	8
4	Blood	High Dependency Unit	Male	Hippokration	16
5	Burn wound	High Dependency Unit	Male	G. Papanikolaou	16
6	Bronchial secretion	Respiratory Failure Unit	Female	G. Papanikolaou	4
7	Urine	Plastic Surgery Unit	Female	G. Papanikolaou	16
8	Bronchial secretion	Cardiology Ward	Male	G. Papanikolaou	16
9	Blood	ICU	Female	Hippokration	16
10	Blood	Obstetrics and Gynecology Unit	Female	Hippokration	4
11	Blood	ICU	Male	Hippokration	32
12	Central venous catheter tip	Internal Medicine Unit	Female	Hippokration	16
13	Urine	Internal Medicine Unit	Male	Hippokration	16
14	Blood	ICU	Male	Hippokration	8
15	Urine	ICU	Male	Hippokration	8
16	Bronchial secretion	Internal Medicine Unit	Female	Hippokration	8
17	Bronchial secretion	Internal Medicine Unit	Female	Hippokration	8
18	Bronchial secretion	ICU	Female	Hippokration	16
19	Bronchial secretion	ICU	Male	Hippokration	4
20	Bronchoalveolar lavage	Neurology Ward	Female	G. Papanikolaou	16
21	Bronchial secretion	Surgical Ward	Female	G. Papanikolaou	8
22	Bronchial secretion	ICU	Male	G. Papanikolaou	4
23	Stool	Respiratory Failure Unit	Female	G. Papanikolaou	16
24	Urine	ICU	Male	G. Papanikolaou	16
25	Bronchial secretion	Plastic Surgery Unit	Female	G. Papanikolaou	16
26	Blood	ICU	Female	G. Papanikolaou	16
27	Blood	Plastic Surgery Unit	Female	G. Papanikolaou	16
28	Urine	ICU	Female	Hippokration	8
29	Blood	Internal Medicine Unit	Female	Hippokration	16
30	Skin lesion	ICU	Male	Hippokration	4
31	Blood	ICU	Male	Hippokration	8
32	Blood	Internal Medicine Unit	Male	Hippokration	16
33	Blood	Surgical Ward	Male	Hippokration	8
34	Blood	ICU	Male	Hippokration	16
35	Central venous catheter tip	ICU	Male	Hippokration	8
36	Bronchial secretion	ICU	Male	Hippokration	4
37	Blood	Internal Medicine Unit	Male	Hippokration	4
38	Bronchial secretion	ICU	Male	Hippokration	8
39	Bronchial secretion	ICU	Female	Hippokration	4
40	Skin lesion	Nephrology Ward	Male	Hippokration	8

Supplementary Table S1. The table presents the isolate identification number, clinical specimen type, hospital unit from which the sample was obtained, patient sex, hospital of origin, and the minimum inhibitory concentration (MIC) of colistin expressed in μg/mL.

**Sequence Type**	**Number of isolates**	***pmrB* A226V (%)**	***pmrB* E210D (%)**	***pmrB* K179M (%)**	***lpxD* E117K (%)**	***lpxC* N287D (%)**	***pmrC* N284D (%)**	***pmrC* V42I. (%)**	***pmrC* R109P (%)**	***pmrC* F150L (%)**	***pmrC* K515T (%)**
ST2	8	7/8 (87.5)	0/8 (0.0)	0/8 (0.0)	7/8 (87.5)	8/8 (100.0)	8/8 (100.0)	8/8 (100.0)	8/8 (100.0)	8/8 (100.0)	8/8 (100.0)
ST1	1	1/1 (100.0)	0/1 (0.0)	0/1 (0.0)	1/1 (100.0)	1/1 (100.0)	1/1 (100.0)	1/1 (100.0)	1/1 (100.0)	1/1 (100.0)	1/1 (100.0)
ST115	1	1/1 (100.0)	0/1 (0.0)	0/1 (0.0)	1/1 (100.0)	0/1 (0.0)	1/1 (100.0)	0/1 (0.0)	0/1 (0.0)	0/1 (0.0)	0/1 (0.0)

Supplementary Table S2. Frequency of variable amino-acid substitutions (present in some but not all typed isolates), stratified by MLST sequence type (ST).

Isolate number	Mutations in the *lpxA, LpxC*, and *LpxD* genes						Mutation in the *pmrA, pmrB*, and *pmrC* genes					
	lpxA Nucleotide	*lpxA* Amino acid	*lpxD* Nucleotide	*lpxD* Amino acid	*lpxC* Nucleotide	*lpxC* Amino acid	*pmrA* Nucleotide	*pmrA* Amino acid	*pmrB* Nucleotide	*pmrB* Amino acid	*pmrC* Nucleotide	*pmrC* Amino acid
1			**G349A**, A642G	E117K	**A859G**, C201T, A552G, C597T, T600C, T666G, G807A	N287D	A120G, C405T, C463T, C633T, T450C		A777G, A786G, A864T, C561A, **C677T**, C709T, C744T	**A226V**	**G124A**, C219T, G246A, T264C, **G326C**, **T450G**, A453C, A711T, T712C, T573C, C645A, G693A, A813G, **A850G**, T936G, A966G, G1060T, G1089A, C1102T, A1110G, A1146G, G1152A, T1164C, T1185G, T1212C, T1242C, G1299A, **A1544C**	**F150L**, **K515T**, **N284D**, **R109P**, **V42I**
2	T93C, C99T, A456G, T495C, A732G		**G349A**, A642G	E117K	**A859G**, C201T, A552G, C597T, T600C, T666G, G807A	N287D	A120G, C405T, C463T, C633T, T450C		A777G, A786G, A864T, C561A, C709T, C744T		**G124A**, C219T, G246A, T264C, **G326C**, **T450G**, A453C, A711T, T712C, T573C, C645A, G693A, A813G, **A850G**, T936G, A966G, G1060T, G1089A, C1102T, A1110G, A1146G, G1152A, T1164C, T1185G, T1212C, T1242C, G1299A, **A1544C**	**F150L**, **K515T**, **N284D**, **R109P**, **V42I**
3	T93C, C99T, A456G, T495C, A732G		**G349A**, A642G	E117K	**A859G**, C201T, A552G, C597T, T600C, T666G, G807A	N287D	A120G, C405T, C463T, C633T, T450C		A777G, A786G, A864T, C561A, **C677T**, C709T, C744T	**A226V**	**G124A**, C219T, G246A, T264C, **G326C**, **T450G**, A453C, A711T, T712C, T573C, C645A, G693A, A813G, **A850G**, T936G, A966G, G1060T, G1089A, C1102T, A1110G, A1146G, G1152A, T1164C, T1185G, T1212C, T1242C, G1299A, **A1544C**	**F150L**, **K515T**, **N284D**, **R109P**, **V42I**
4	T93C, C99T, A456G, T495C, A732G		**G349A**	E117K	**A859G**, C201T, A552G, C597T, T600C, T666G, G807A	N287D	A120G, C405T, C463T, C633T, T450C		A777G, A786G, A864T, C561A, **C677T**, C709T, C744T	**A226V**	**G124A**, C219T, G246A, T264C, **G326C**, **T450G**, A453C, A711T, T712C, T573C, C645A, G693A, A813G, **A850G**, T936G, A966G, G1060T, G1089A, C1102T, A1110G, A1146G, G1152A, T1164C, T1185G, T1212C, T1242C, G1299A, **A1544C**	**F150L**, **K515T**, **N284D**, **R109P**, **V42I**
5	T93C, C99T, A456G, G558T		**G349A**, A642G	E117K	**A859G**, C201T, A252C, C355T, A552G, G660A, G699A	N287D	A120G, C405T, C463T, C633T, T450C		A777G, A786G, A864T, C561A, **C677T**, C709T, C744T	**A226V**	**G124A**, C219T, G246A, T264C, **G326C**, **T450G**, A453C, A711T, T712C, T573C, C645A, G693A, A813G, **A850G**, T936G, A966G, G1060T, G1089A, C1102T, A1110G, A1146G, G1152A, T1164C, T1185G, T1212C, T1242C, G1299A, **A1544C**	**F150L**, **K515T**, **N284D**, **R109P**, **V42I**
6	T93C, C99T, A456G, T495C, A732G		**G349A**, A642G	E117K	**A859G**, C201T, A552G, C597T, T600C, T666G, G807A	N287D	A120G, C405T, C463T, C633T, T450C		A777G, A786G, A864T, C561A, **C677T**, C709T, C744T	**A226V**	**G124A**, C219T, G246A, T264C, **G326C**, **T450G**, A453C, A711T, T712C, T573C, C645A, G693A, A813G, **A850G**, T936G, A966G, G1060T, G1089A, C1102T, A1110G, A1146G, G1152A, T1164C, T1185G, T1212C, T1242C, G1299A, **A1544C**	**F150L**, **K515T**, **N284D**, **R109P**, **V42I**
7	T93C, C99T, A456G, T495C, A732G		**G349A**, A642G	E117K	**A859G**, C201T, A552G, C597T, T600C, T666G, G807A	N287D	A120G, C405T, C463T, C633T, T450C		A777G, A786G, A864T, C561A, **C677T**, C709T, C744T	**A226V**	**G124A**, C219T, G246A, T264C, **G326C**, **T450G**, A453C, A711T, T712C, T573C, C645A, G693A, A813G, **A850G**, T936G, A966G, G1060T, G1089A, C1102T, A1110G, A1146G, G1152A, T1164C, T1185G, T1212C, T1242C, G1299A, **A1544C**	**F150L**, **K515T**, **N284D**, **R109P**, **V42I**
8	T93C, C99T, A456G, T495C, A732G		**G349A**, A642G	E117K	**A859G**, C201T, A552G, C597T, T600C, T666G, G807A	N287D	A120G, C405T, C463T, C633T, T450C		A777G, A786G, A864T, C561A, **C677T**, C709T, C744T	**A226V**	**G124A**, C219T, G246A, T264C, **G326C**, **T450G**, A453C, A711T, T712C, T573C, C645A, G693A, A813G, **A850G**, T936G, A966G, G1060T, G1089A, C1102T, A1110G, A1146G, G1152A, T1164C, T1185G, T1212C, T1242C, G1299A, **A1544C**	**F150L**, **K515T**, **N284D**, **R109P**, **V42I**
9	T93C, C99T, A456G, G558T				**A859G**, C201T, A252C, C355T, A552G, G660A, G699A	N287D	A192G, A75G		A777G, A786G, A864T, C561A, **C677T**, C709T, C744T	**A226V**	T279C, G285C, C291A, T297G, C325T, **A343G**, G510A, C645A, G693A, A711T, T712C, A813G, **A850G**, T936G, C960T, T1086C, G1089A, C1102T, A1110G, T1122C, A1125G, A1146G, T1164C, T1185G, T1212C, T1242C, G1299A, **A1448G**	**H483R**, **I115V**, **N284D**
10	T93C, C99T, A456G, T495C, A732G		**G349A**, A642G	E117K	**A859G**, C201T, A552G, C597T, T600C, T666G, G807A	N287D	A120G, C405T, C463T, C633T, T450C		A777G, A786G, A864T, C561A, **C677T**, C709T, C744T	**A226V**	**G124A**, C219T, G246A, T264C, **G326C**, **T450G**, A453C, A711T, T712C, T573C, C645A, G693A, A813G, **A850G**, T936G, A966G, G1060T, G1089A, C1102T, A1110G, A1146G, G1152A, T1164C, T1185G, T1212C, T1242C, G1299A, **A1544C**	**F150L**, **K515T**, **N284D**, **R109P**, **V42I**
11	T93C, C99T, A456G, T495C, A732G		**G349A**, A642G	E117K	**A859G**, C201T, A552G, C597T, T600C, T666G, G807A	N287D	A120G, C405T, C463T, C633T, T450C		C477T, C633A, C709T, C738T, C912T, T688C		**G124A**, C219T, G246A, T264C, **G326C**, **T450G**, A453C, A711T, T712C, T573C, C645A, G693A, A813G, **A850G**, T936G, A966G, G1060T, G1089A, C1102T, A1110G, A1146G, G1152A, T1164C, T1185G, T1212C, T1242C, G1299A, **A1544C**	**F150L**, **K515T**, **N284D**, **R109P**, **V42I**
12	A456G		T150C, T682C, A687G, C732T		**A859G**, C201T, A252C, A552G, C597T, T600C, T681A, C711T, T718C	N287D	A120G, C405T, C463T, C633T, T450C		A777G, A786G, A864T, C561A, **C677T**, C709T, C744T	**A226V**	T199C, C219T, C240T, T264C, C313T, A343G, A381G, C432T, C459T, G600A, C642A, C645A, G693A, A711T, T712C, A813G, **A850G**, T936G, C960T, **T977C**, T1086C, G1089A, C1102T, A1110G, A1146G, T1164C, T1185G, T1212C, T1242C, C1282T, G1299A	**F150L**, **K515T**, **N284D**, **R109P**, **V42I**
13	T93C, C99T, A456G, T495C, A732G		**G349A**, A642G	E117K	**A859G**, C201T, A552G, C597T, T600C, T666G, G807A	N287D	A120G, C405T, C463T, C633T, T450C		A777G, A786G, A864T, C561A, **C677T**, C709T, C744T	**A226V**	**G124A**, C219T, G246A, T264C, **G326C**, **T450G**, A453C, A711T, T712C, T573C, C645A, G693A, A813G, **A850G**, T936G, A966G, G1060T, G1089A, C1102T, A1110G, A1146G, G1152A, T1164C, T1185G, T1212C, T1242C, G1299A, **A1544C**	**F150L**, **K515T**, **N284D**, **R109P**, **V42I**
14	T93C, C99T, A456G, T495C, A732G		**G349A**, A642G	E117K	**A859G**, C201T, A552G, C597T, T600C, T666G, G807A	N287D	A120G, C405T, C463T, C633T, T450C		C477T, C633A, C709T, C738T, C912T, G795A, T688C, T747C		**G124A**, C219T, G246A, T264C, **G326C**, **T450G**, A453C, A711T, T712C, T573C, C645A, G693A, A813G, **A850G**, T936G, A966G, G1060T, G1089A, C1102T, A1110G, A1146G, G1152A, T1164C, T1185G, T1212C, T1242C, G1299A, **A1544C**	**F150L**, **K515T**, **N284D**, **R109P**, **V42I**
15	T93C, C99T, A456G, T495C, A732G		**G349A**, A642G	E117K			A120G, C405T, C463T, C633T, T450C		A777G, A786G, A864T, C561A, **C677T**, C709T, C744T	**A226V**	**G124A**, C219T, G246A, T264C, **G326C**, **T450G**, A453C, A711T, T712C, T573C, C645A, G693A, A813G, **A850G**, T936G, A966G, G1060T, G1089A, C1102T, A1110G, A1146G, G1152A, T1164C, T1185G, T1212C, T1242C, G1299A, **A1544C**	**F150L**, **K515T**, **N284D**, **R109P**, **V42I**

16	T93C, C99T, A456G, T495C, A732G		**G349A**, A642G	E117K	**A859G**, C201T, A552G, C597T, T600C, T666G, G807A	N287D	A120G, C405T, C463T, C633T, T450C		A777G, A786G, A864T, C561A, **C677T**, C709T, C744T	**A226V**	**G124A**, C219T, G246A, T264C, **G326C**, **T450G**, A453C, A711T, T712C, T573C, C645A, G693A, A813G, **A850G**, T936G, A966G, G1060T, G1089A, C1102T, A1110G, A1146G, G1152A, T1164C, T1185G, T1212C, T1242C, G1299A, **A1544C**	**F150L**, **K515T**, **N284D**, **R109P**, **V42I**
17	T93C, C99T, A456G, G558T				**A859G**, C201T, A252C, C355T, A552G, G660A, G699A	N287D	A120G, C405T, C463T, C633T, T450C		A777G, A786G, A864T, C561A, **C677T**, C709T, C744T	**A226V**	**G124A**, C219T, G246A, T264C, **G326C**, **T450G**, A453C, A711T, T712C, T573C, C645A, G693A, A813G, **A850G**, T936G, A966G, G1060T, G1089A, C1102T, A1110G, A1146G, G1152A, T1164C, T1185G, T1212C, T1242C, G1299A, **A1544C**	**F150L**, **K515T**, **N284D**, **R109P**, **V42I**
18	T93C, C99T, A456G, T495C, A732G		**G349A**, A642G	E117K	**A859G**, C201T, A552G, C597T, T600C, T666G, G807A	N287D	A120G, C405T, C463T, C633T, T450C		A777G, A786G, A864T, C561A, **C677T**, C709T, C744T	**A226V**	**G124A**, C219T, G246A, T264C, **G326C**, **T450G**, A453C, A711T, T712C, T573C, C645A, G693A, A813G, **A850G**, T936G, A966G, G1060T, G1089A, C1102T, A1110G, A1146G, G1152A, T1164C, T1185G, T1212C, T1242C, G1299A, **A1544C**	**F150L**, **K515T**, **N284D**, **R109P**, **V42I**
19	T93C, C99T, A456G, T495C, A732G		**G349A**, A642G	E117K	**A859G**, C201T, A552G, C597T, T600C, T666G, G807A	N287D	A120G, C405T, C463T, C633T, T450C		A468G, A480G, **A537G**, A540G, A552G, A555G, A570G, A573G, A597G, A786T, A786T, A825C, A864C, A864T, A873G, A888T, C543T, C549T, C556T, C558A, C561A, C609T, C633T, C672T, C691A, C709T, C733T, C744T, C768T, C852T, C912T, C915T, **G628C**, G645A, G702T, G783A, G801A, G855A, G900T, G918A, T603C, T624G, T666C, T688C, T807G, T870C, T897C, T921C	**E210D**, **K179M**	**G124A**, C219T, G246A, T264C, **G326C**, **T450G**, A453C, A711T, T712C, T573C, C645A, G693A, A813G, **A850G**, T936G, A966G, G1060T, G1089A, C1102T, A1110G, A1146G, G1152A, T1164C, T1185G, T1212C, T1242C, G1299A, **A1544C**	**F150L**, **K515T**, **N284D**, **R109P**, **V42I**
20							A120G, C405T, C463T, C633T, T450C		A777G, A786G, A864T, C561A, **C677T**, C709T, C744T	**A226V**	**G124A**, C219T, G246A, T264C, **G326C**, **T450G**, A453C, A711T, T712C, T573C, C645A, G693A, A813G, **A850G**, T936G, A966G, G1060T, G1089A, C1102T, A1110G, A1146G, G1152A, T1164C, T1185G, T1212C, T1242C, G1299A, **A1544C**	**F150L**, **K515T**, **N284D**, **R109P**, **V42I**
21	T93C, C99T, A456G, T495C, A732G		**G349A**, A642G	E117K	**A859G**, C201T, A552G, C597T, T600C, T666G, G807A	N287D	A120G, C405T, C463T, C633T, T450C		A777G, A786G, A864T, C561A, **C677T**, C709T, C744T	**A226V**	**G124A**, C219T, G246A, T264C, **G326C**, **T450G**, A453C, A711T, T712C, T573C, C645A, G693A, A813G, **A850G**, T936G, A966G, G1060T, G1089A, C1102T, A1110G, A1146G, G1152A, T1164C, T1185G, T1212C, T1242C, G1299A, **A1544C**	**F150L**, **K515T**, **N284D**, **R109P**, **V42I**
22	T93C, C99T, A456G, T495C, A732G		**G349A**, A642G	E117K			A120G, C405T, C463T, C633T, T450C		A777G, A786G, A864T, C561A, **C677T**, C709T, C744T	**A226V**	**G124A**, C219T, G246A, T264C, **G326C**, **T450G**, A453C, A711T, T712C, T573C, C645A, G693A, A813G, **A850G**, T936G, A966G, G1060T, G1089A, C1102T, A1110G, A1146G, G1152A, T1164C, T1185G, T1212C, T1242C, G1299A, **A1544C**	**F150L**, **K515T**, **N284D**, **R109P**, **V42I**
23	T93C, C99T, A456G, T495C, A732G		**G349A**, A642G	E117K	**A859G**, C201T, A552G, C597T, T600C, T666G, G807A	N287D	A120G, C405T, C463T, C633T, T450C		A777G, A786G, A864T, C561A, **C677T**, C709T, C744T	**A226V**	**G124A**, C219T, G246A, T264C, **G326C**, **T450G**, A453C, A711T, T712C, T573C, C645A, G693A, A813G, **A850G**, T936G, A966G, G1060T, G1089A, C1102T, A1110G, A1146G, G1152A, T1164C, T1185G, T1212C, T1242C, G1299A, **A1544C**	**F150L**, **K515T**, **N284D**, **R109P**, **V42I**
24	T93C, C99T, A456G, T495C, A732G		**G349A**, A642G	E117K	**A859G**, C201T, A552G, C597T, T600C, T666G, G807A	N287D	A120G, C405T, C463T, C633T, T450C		A777G, A786G, A864T, C561A, **C677T**, C709T, C744T, **821_822ins[ACGATT]**	**273_274ins [LA]**, **A226V**	**G124A**, C219T, G246A, T264C, **G326C**, **T450G**, A453C, A711T, T712C, T573C, C645A, G693A, A813G, **A850G**, T936G, A966G, G1060T, G1089A, C1102T, A1110G, A1146G, G1152A, T1164C, T1185G, T1212C, T1242C, G1299A, **A1544C**	**F150L**, **K515T**, **N284D**, **R109P**, **V42I**
25			**G349A**, A642G	E117K			A120G, C405T, C463T, C633T, T450C		A777G, A786G, A864T, C561A, **C677T**, C709T, C744T	**A226V**	**G124A**, C219T, G246A, T264C, **G326C**, **T450G**, A453C, A711T, T712C, T573C, C645A, G693A, A813G, **A850G**, T936G, A966G, G1060T, G1089A, C1102T, A1110G, A1146G, G1152A, T1164C, T1185G, T1212C, T1242C, G1299A, **A1544C**	**F150L**, **K515T**, **N284D**, **R109P**, **V42I**
26			**G349A**, A642G	E117K	**A859G**, C201T, A552G, C597T, T600C, T666G, G807A	N287D	A120G, C405T, C463T, C633T, T450C		A777G, A786G, A864T, C561A, **C677T**, C709T, C744T, **C824A**	**A226V**, **A275E**	**G124A**, C219T, G246A, T264C, **G326C**, **T450G**, A453C, A711T, T712C, T573C, C645A, G693A, A813G, **A850G**, T936G, A966G, G1060T, G1089A, C1102T, A1110G, A1146G, G1152A, T1164C, T1185G, T1212C, T1242C, G1299A, **A1544C**	**F150L**, **K515T**, **N284D**, **R109P**, **V42I**
27	T93C, C99T, A456G, T495C, A732G		**G349A**, A642G	E117K	**A859G**, C201T, A252C, C355T, A552G, G660A, T666G, G699A	N287D	A120G, C405T, C463T, C633T, T450C		A777G, A786G, A864T, C561A, **C677T**, C709T, C744T	**A226V**	**G124A**, C219T, G246A, T264C, **G326C**, **T450G**, A453C, A711T, T712C, T573C, C645A, G693A, A813G, **A850G**, T936G, A966G, G1060T, G1089A, C1102T, A1110G, A1146G, G1152A, T1164C, T1185G, T1212C, T1242C, G1299A, **A1544C**	**F150L**, **K515T**, **N284D**, **R109P**, **V42I**
28	T93C, C99T, A456G, T495C, A732G		**G349A**, A642G	E117K	**A859G**, C201T, A552G, C597T, T600C, T666G, G807A	N287D	A120G, C405T, C463T, C633T, T450C		C477T, C633A, C709T, C738T, C912T, T688C		**G124A**, C219T, G246A, T264C, **G326C**, **T450G**, A453C, A711T, T712C, T573C, C645A, G693A, A813G, **A850G**, T936G, A966G, G1060T, G1089A, C1102T, A1110G, A1146G, G1152A, T1164C, T1185G, T1212C, T1242C, G1299A, **A1544C**	**F150L**, **K515T**, **N284D**, **R109P**, **V42I**
29	T93C, C99T, A456G, T495C, A732G		**G349A**, A642G	E117K	**A859G**, C201T, A552G, C597T, T600C, T666G, G807A	N287D	A120G, C405T, C463T, C633T, T450C		A777G, A786G, A864T, C561A, **C677T**, C709T, C744T	**A226V**	**G124A**, C219T, G246A, T264C, **G326C**, **T450G**, A453C, A711T, T712C, T573C, C645A, G693A, A813G, **A850G**, T936G, A966G, G1060T, G1089A, C1102T, A1110G, A1146G, G1152A, T1164C, T1185G, T1212C, T1242C, G1299A, **A1544C**	**F150L**, **K515T**, **N284D**, **R109P**, **V42I**

30			**G349A**, A642G	E117K	**A859G**, C201T, A252C, A552G, C597T, T600C, T681A, C711T, T718C	N287D	A120G, C405T, C463T, C633T, T450C		A777G, A786G, A864T, C561A, **C677T**, C709T, C744T	**A226V**	**G124A**, C219T, G246A, T264C, **G326C**, **T450G**, A453C, A711T, T712C, T573C, C645A, G693A, A813G, **A850G**, T936G, A966G, G1060T, G1089A, C1102T, A1110G, A1146G, G1152A, T1164C, T1185G, T1212C, T1242C, G1299A, **A1544C**	**F150L**, **K515T**, **N284D**, **R109P**, **V42I**
31	T93C, C99T, A456G, T495C, A732G		**G349A**, A642G	E117K	**A859G**, C201T, A552G, C597T, T600C, G807A	N287D	A120G, C405T, C463T, C633T, T450C		A777G, A786G, A864T, C561A, **C677T**, C709T, C744T	**A226V**	**G124A**, C219T, G246A, T264C, **G326C**, **T450G**, A453C, A711T, T712C, T573C, C645A, G693A, A813G, **A850G**, T936G, A966G, G1060T, G1089A, C1102T, A1110G, A1146G, G1152A, T1164C, T1185G, T1212C, T1242C, G1299A, **A1544C**	**F150L**, **K515T**, **N284D**, **R109P**, **V42I**
32			**G349A**, A642G	E117K	**A859G**, C201T, A552G, C597T, T600C, T666G, G807A	N287D	A120G, C405T, C463T, C633T, T450C		A468G, A480G, **A537G**, A540G, A552G, A555G, A570G, A573G, A597G, A786T, A786T, A825C, A864C, A864T, A873G, A888T, C543T, C549T, C556T, C558A, C561A, C609T, C633T, C672T, C691A, C709T, C733T, C744T, C768T, C852T, C912T, C915T, G628C, G645A, G702T, G783A, G801A, G855A, G900T, G918A, T603C, T624G, T666C, T688C, T807G, T870C, T897C, T921C	**E210D**, **K179M**	**G124A**, C219T, G246A, T264C, **G326C**, **T450G**, A453C, A711T, T712C, T573C, C645A, G693A, A813G, **A850G**, T936G, A966G, G1060T, G1089A, C1102T, A1110G, A1146G, G1152A, T1164C, T1185G, T1212C, T1242C, G1299A, **A1544C**	**F150L**, **K515T**, **N284D**, **R109P**, **V42I**
33	T93C, C99T, A456G, T495C, A732G		**G349A**, A642G	E117K	**A859G**, C201T, A552G, C597T, T600C, T666G, G807A	N287D	A120G, C405T, C463T, C633T, T450C		C477T, C633A, C709T, C738T, C912T, T688C		**G124A**, C219T, G246A, T264C, **G326C**, **T450G**, A453C, A711T, T712C, T573C, C645A, G693A, A813G, **A850G**, T936G, A966G, G1060T, G1089A, C1102T, A1110G, A1146G, G1152A, T1164C, T1185G, T1212C, T1242C, G1299A, **A1544C**	**F150L**, **K515T**, **N284D**, **R109P**, **V42I**
34	T93C, C99T, A456G, T495C, A732G		**G349A**, A642G	E117K	**A859G**, C201T, A552G, C597T, T600C, T666G, G807A	N287D	A120G, C405T, C463T, C633T, T450C		C477T, C633A, C709T, C738T, C912T, T688C		**G124A**, C219T, G246A, T264C, **G326C**, **T450G**, A453C, A711T, T712C, T573C, C645A, G693A, A813G, **A850G**, T936G, A966G, G1060T, G1089A, C1102T, A1110G, A1146G, G1152A, T1164C, T1185G, T1212C, T1242C, G1299A, **A1544C**	**F150L**, **K515T**, **N284D**, **R109P**, **V42I**
35	T93C, C99T, A456G, T495C, A732G				**A859G**, C201T, A252C, C355T, A552G, G660A, G699A	N287D	A120G, C405T, C463T, C633T, T450C		C477T, C633A, C709T, C738T, C912T, T688C		**G124A**, C219T, G246A, T264C, **G326C**, **T450G**, A453C, A711T, T712C, T573C, C645A, G693A, A813G, **A850G**, T936G, A966G, G1060T, G1089A, C1102T, A1110G, A1146G, G1152A, T1164C, T1185G, T1212C, T1242C, G1299A, **A1544C**	**F150L**, **K515T**, **N284D**, **R109P**, **V42I**
36	A456G				**A859G,** A552G,	N287D	A120G, C405T, C463T, C633T, T450C		C477T, C633A, C709T, C738T, C912T, T688C		**G124A**, C219T, G246A, T264C, **G326C**, **T450G**, A453C, A711T, T712C, T573C, C645A, G693A, A813G, **A850G**, T936G, A966G, G1060T, G1089A, C1102T, A1110G, A1146G, G1152A, T1164C, T1185G, T1212C, T1242C, G1299A, **A1544C**	**F150L**, **K515T**, **N284D**, **R109P**, **V42I**
37	A456G				**A859G,** A552G	N287D	A120G, C405T, C463T, C633T, T450C		C477T, C633A, C709T, C738T, C912T, T688C		**G124A**, C219T, G246A, T264C, **G326C**, **T450G**, A453C, A711T, T712C, T573C, C645A, G693A, A813G, **A850G**, T936G, A966G, G1060T, G1089A, C1102T, A1110G, A1146G, G1152A, T1164C, T1185G, T1212C, T1242C, G1299A, **A1544C**	**F150L**, **K515T**, **N284D**, **R109P**, **V42I**
38	A456G				**A859G,** A552G	N287D	A120G, C405T, C463T, C633T, T450C		C477T, C633A, C709T, C738T, C912T, T688C		**G124A**, C219T, G246A, T264C, **G326C**, **T450G**, A453C, A711T, T712C, T573C, C645A, G693A, A813G, **A850G**, T936G, A966G, G1060T, G1089A, C1102T, A1110G, A1146G, G1152A, T1164C, T1185G, T1212C, T1242C, G1299A, **A1544C**	**F150L**, **K515T**, **N284D**, **R109P**, **V42I**
39	A456G				**A859G,** A552G	N287D	A120G, C405T, C463T, C633T, T450C		C477T, C633A, C709T, C738T, C912T, T688C		**G124A**, C219T, G246A, T264C, **G326C**, **T450G**, A453C, A711T, T712C, T573C, C645A, G693A, A813G, **A850G**, T936G, A966G, G1060T, G1089A, C1102T, A1110G, A1146G, G1152A, T1164C, T1185G, T1212C, T1242C, G1299A, **A1544C**	**F150L**, **K515T**, **N284D**, **R109P**, **V42I**
40	A456G				**A859G,** A552G	N287D	A120G, C405T, C463T, C633T, T450C		C477T, C633A, C709T, C738T, C912T, T688C		**G124A**, C219T, G246A, T264C, **G326C**, **T450G**, A453C, A711T, T712C, T573C, C645A, G693A, A813G, **A850G**, T936G, A966G, G1060T, G1089A, C1102T, A1110G, A1146G, G1152A, T1164C, T1185G, T1212C, T1242C, G1299A, **A1544C**	**F150L**, **K515T**, **N284D**, **R109P**, **V42I**

Table S3. Nucleotide and amino acid mutations detected in *lpxA*, *lpxC*, *lpxD*, *pmrA*, *pmrB*, and *pmrC* genes among the 40 clinical isolates. Nucleotide substitutions and corresponding amino acid changes identified in the lipid A biosynthesis genes (*lpxA, lpxC, lpxD*) and the two-component regulatory system genes (*pmrA, pmrB*), as well as the lipid A modification gene (pmrC), are presented for each isolate. Mutations are annotated according to coding DNA position, and amino acid substitutions are indicated using standard one-letter notation. Insertions are reported at the nucleotide and protein level where applicable. Empty cells indicate absence of detected mutations in the respective gene.

Gene		Primer sequence (5’ → 3’)	Product size (bp)	References
lpxA	FW	TGAAGCATTAGCTCAAGTTT	1181	[38]
	RV	GTCAGCAAATCAATACAAGA		
lpxD	FW	CAAAGTATGAATACAACTTTTGAG	1163	[38]
	RV	GTCAATGGCACATCTGCTAAT		
lpxC	FW	TGAAGATGACGTTCCTGCAA	1501	[38]
	RV	TGGTGAAAATCAGGCAATGA		
pmrA	FW	ATGACAAAAATCTTGATGATTGAAGAT	653	[42]
	RV	TTATGATTGCCCCAAACGGTAG		
pmrB	FW	GTGCATTATTCATTAAAAAAAC	1335	[42]
	RV	TCACGCTCTTGTTTCATGTA		
pmrB2	FW	GGTTCGTGAAGCTTTCG	529	[42]
	RV	CCTAAATCGATTTCTTTTTG		
pmrC	FW	ATGTTTAATCTCATTATAGCCA	1583	[42]
	RV	TTAGTTTACATGGGCACAA		
pmrC2	FW	GGTTGTTATTGAAGAAAGTAT	495	[42]
	RV	TCAATCCAAGTCACTTGGTAAC		
cpn60	FW	ACTGTACTTGCTCAAGC	405	[52]
	RV	TTCAGCGATGATAAGAAGTGG		
fusA	FW	ATCGGTATTTCTGCKCACATYGAT	633	[52]
	RV	CCAACATACKYTGWACACCTTTGTT		
gltA	FW	AATTTACAGTGGCACATTAGGTCCC	483	[52]
	RV	GCAGAGATACCAGCAGAGATACACG		
pyrG	FW	GGTGTTGTTTCATCACTAGGWAAAGG	297	[52]
	RV	ATAAATGGTAAAGAYTCGATRTCACCMA		
recA	FW	CCTGAATCTTCYGGTAAAAC	372	[52]
	RV	GTTTCTGGGCTGCCAAACATTAC		
rplB	FW	GTAGAGCGTATTGAATACGATCCTAACC	330	[52]
	RV	CACCACCACCRTGYGGGTGATC		
rpoB	FW	GGCGAAATGGCRGARAACCA	456	[52]
	RV	GARTCYTCGAAGTTGTAACC		

Supplementary Table S4. Primers (5'→3') and expected PCR product sizes (bp) used for the amplification of the *lpxA*, *lpxD, lpxC, pmrA*, *pmrB*, *pmrB2*, *pmrC*, and *pmrC2* genes, as well as the seven housekeeping genes *cpn60*, *fusA*, *gltA*, *pyrG*, *recA*, *rplB*, and *rpoB* included in the MLST scheme for *A. baumannii*. Primers for *lpxA*, *lpxD*, and *lpxC* were designed according to Moffatt et al. [38], whereas primers for *pmrA*, *pmrB*, *pmrB2*, *pmrC*, and *pmrC2* were designed according to Beceiro et al. [42]. Primers for the MLST loci were obtained from the PubMLST database [52]. The same primers were used for bidirectional Sanger sequencing of the PCR products.
